# Earl Beatty and the Nation's Duty

**Published:** 1920-03-20

**Authors:** 


### THE MEN OF THE GREAT SEA SERVICES.

### Earl Beatty and the Nation's Duty

The Seamen's Hospital Society has added another to its famous annual meetings. At the Annual Court of Governors, held at the Queen's Hall on March 11, the hall, vast as it is, was almost full, and one of the speakers remarked that next year they would have to hold the Annual Court in Hyde Park! It was probably one of the largest hospital meetings that has ever been held. About 2,000 people were present, and a feature of the occasion was the band of the Royal Marine Artillery, which attended by favour of General Sir David Mercer, Commandant of the Royal Marines. The Right Hon. Earl Beatty, O.M., Admiral of the Fleet, presided.

A Year of Activity.

**Sir Perceval Nairne,** Chairman of the Committee, in opening the proceedings, said that the year 1919 was a very active one in the Seamen's Hospital Society. The principal development which was brought about in that year was the acquisition of the Endsleigh Gardens property as a hospital for persons who had been serving in his Majesty's Forces, and in the course of that service had contracted tropical disease. With the very great assistance of the British Red Cross Society and the Order of St. John, the Seamen's Hospital Society was placed in a position to acquire that property, and the hospital was now open for the treatment of patients. It had also been found possible within the building of which the hospital formed part to provide accommodation for the London School of Tropical Medicine, a branch of the operations of the Seamen's Hospital which was started in 1898. During the year the Angas Home at Cudham had been placed in full working order; and, furthermore, the Society had succeeded in acquiring a property on the borders of Hampshire and Surrey for the treatment of tuberculous seamen patients. The thanks of the Society were greatly due to Lord Inchcape, who had established a fund for the fitting-out and endowment of this sanatorium.

The Royal Navy and the Mercantile Marine.

**Earl Beatty,** whose speech was delayed by the unsuccessful efforts of a photographer to get a flashlight picture, the Earl allowing him "One more shot," said that the institution would commend itself to the hearts of all citizens of the British Empire. He, as a member of the Sea Service, felt very much honoured on being asked to preside over the Annual Court of Governors, and thereby to have the privilege of saying something on behalf of an institution which was doing so much for his comrades of the sea. The brotherhood of the sea had always been a very real and live thing, and the late war, with all its tragedies by water, had welded the Royal Navy and the Mercantile Marine together in a final bond. This Society looked after that great sea service. For the past hundred years it had been a boon and a blessing to sailors. It commenced its operations with the battleship called the *Grampus*, lent to the Society by the Government of the day, and this was replaced by another, the *Dreadnought*, which name had been associated with the Society ever since. This vessel was followed by the *Caledonia*, renamed the *Dreadnought*, and thus the work went on until 1870, when the old infirmary at Greenwich was placed at the Society's disposal. To-day the Society had extended until it supported three hospitals, one convalescent home, and two dispensaries, required an income of £40,000 per annum, and helped about 20,000 British sailors a year. During the war the work of the Society was not confined to the men of the merchant service, but many thousands of naval ratings were treated in the wards. It was now looking forward to the establishment of a sanatorium, and for that especially he would like to plead.

The Penalties of the Sailor's Calling.

The existing sanatorium accommodation in this country was inadequate for the general population, and those who did gain access to sanatoriums did so by reason of being domiciled in some particular neighbourhood, where the local authorities had the power to pay for their treatment and maintenance. The sailor was on a different footing. His duties called him from home; he had none of the advantages of a permanent domicile, and he was seldom able to obtain proper care and treatment. He wondered how many of the ladies and gentlemen present that afternoon realised the conditions under which sailors lived on board ship, more especially in the merchant service, confined in narrow spaces, frequently battened down for days without any means of ventilation, and even when in harbour enjoying none of the privacy to which all men were entitled, or of the comforts which were to be expected in the humblest dwelling. "We are an island race," said Lord Beatty in conclusion, "and it is a platitude which will bear repeating that by the sea we exist. This war more than all wars has proved it. Therefore we cannot afford to ignore the men of the great sea service, whether they belong to the Mercantile Marine or the Royal Navy. It is not charity we ask for from you. It is a duty. And it is a duty not to them, but to ourselves and our children." (Applause.)

**Captain A. W. Clarke, C.B.E.,** Deputy Chairman of the Committee of Management, seconded the adoption of the annual report (which Lord Beatty had proposed), and said that much had been heard lately of the strain upon hospitals owing to the increased cost of living and other matters. Various remedies had been put forward, and there were those who declared that the day of the voluntary hospital was drawing to an end, that the sun of voluntary support, which had warmed not only receiver but giver, was about to set. So far as the Seamen's Hospital Society was concerned, this was neither its experience nor its apprehension. He firmly believed that the voluntary spirit which was so characteristic of the British peoples was not weary of doing well. The cherishing of that spirit up to the time of the Great War enabled us to call upon it in the most successful manner, a manner without precedent in the history of the world. He hoped that the hospital supporters would come forward with a still larger generosity to maintain the voluntary principle in its foremost stronghold, the hospitals of old England.

Money Saved to the Country.

**Dr. Addison,** the Minister of Health, proposed a resolution, which he confessed he had not read until that moment, that the officers of the Society whose names were printed in the annual report be elected for the ensuing year. He remarked that there was associated with the hospital whose interests they were advocating that afternoon a school of tropical medicine. Our people realised very little of what it meant for this country to be kept free to the extent it was from a large number of the frightful

**The Men of the Great Sea Service**—(*continued*).

diseases which were at the present time ravaging great tracts of country in Europe. They never stayed to inquire, and even less seldom did they seek to explore, what sources of information were employed in order to enable us to develop our preventive services. In no small measure did the nation at this day owe its immunity from a large number of diseases to the work which had been carried on by devoted men connected with that hospital and tropical school. If the work of such men were to be assessed in terms of money saved to the country, it would be a sum that would provide more sanatoriums than could be dealt with.

The Proposed Sanatorium.

Proceeding to speak of the projected sanatorium, Dr. Addison said that nationally we found ourselves last year short of eight thousand beds in sanatoriums, and it had therefore been decided to double the amount contributed nationally to provide the necessary beds. He was glad to announce that these had been increased by nearly five thousand during the last six months. As for the sanatorium which the Society was proposing, he understood that occupational measures would form a part of its routine. He regarded it as very important that men in sanatoriums should have something to occupy their minds and fingers. It might be that many of the men taken in hand would not be able to return to the ships, and, indeed, they were now finding that in sanatorium cases they had to go a step further, and to see that the men, on being discharged from the sanatorium, had a chance to live in surroundings which would not undo what had been done for them in the institution. "So far as you want help from us," said Dr. Addison, turning to the members of the Committee on the platform, "you have only got to come along, and we will do what we can."

The State and the Voluntary Principle.

In a final reference to the continuance of the voluntary principle, the Minister of Health said that he did not believe in any necessary antagonism between State assistance and the encouragement of voluntary institutions. He remembered that when it was decided to give some Government help to medical schools there was an outcry in certain quarters that it would be undermining their independence. But their independence had not been undermined. On the contrary, it was most vigorously asserted, as anyone like himself knew quite well. And he saw no reason to fear, if they supported, as he hoped they would, generously and whole-heartedly this great endeavour that it would be found hereafter that any withering blight would spread over it as a consequence of undue interference from the State.

**Captain Ian Hamilton Benn, C.B., D.S.O., M.P.**, seconded the resolution, and, in doing so, said that he wished to acknowledge the obligation which the general body of the Governors were under towards certain individuals in particular—he named Sir Perceval Nairne (the Chairman of the Committee of Management), Captain Clarke (the Deputy-Chairman), and Sir Henry Jackson—who constantly attended the meetings and interested themselves so thoroughly in every detail of the working of the Society. He also acknowledged the great assistance which the Society had received from Lord Inchcape in connection with the sanatorium, and this, coupled with what had just been promised them from Dr. Addison, put them in great heart that day. Bramshott Place, for which the Committee had entered into negotiations, had been approved by the Minister of Health, and Lord Inchcape was inviting contributions to a subscription list, which he had headed with £10,000.

The resolution was carried, and the proceedings concluded with a heartily accorded vote of thanks to Lord Beatty, proposed by Sir John Wimble and seconded by Sir William Bennett.

